# Mass Spectrometry and Structural Biology Techniques in the Studies on the Coronavirus-Receptor Interaction

**DOI:** 10.3390/molecules25184133

**Published:** 2020-09-10

**Authors:** Danuta Witkowska

**Affiliations:** Institute of Health Sciences, Opole University, Katowicka 68, 45-060 Opole, Poland; danuta.witkowska@uni.opole.pl

**Keywords:** spike protein-ACE2 interaction, MS, structural techniques, glycosylation, SARS coronavirus

## Abstract

Mass spectrometry and some other biophysical methods, have made substantial contributions to the studies on severe acute respiratory syndrome coronavirus 2 (SARS-CoV-2) and human proteins interactions. The most interesting feature of SARS-CoV-2 seems to be the structure of its spike (S) protein and its interaction with the human cell receptor. Mass spectrometry of spike S protein revealed how the glycoforms are distributed across the S protein surface. X-ray crystallography and cryo-electron microscopy made huge impact on the studies on the S protein and ACE2 receptor protein interaction, by elucidating the three-dimensional structures of these proteins and their conformational changes. The findings of the most recent studies in the scope of SARS-CoV-2-Human protein-protein interactions are described here.

## 1. Introduction

Emerging infectious diseases caused by severe acute respiratory syndrome coronaviruses (SARS-CoV and SARS-CoV-2) present a tremendous threat to international public health [1]. The risk to the global population brought by the Middle East respiratory syndrome (MERS-CoV) [2] is deemed to be much lower. According to the World Health Organization (WHO), the fatality rate of MERS-CoV is quite high (36%) [3,4]; however, the virus does not seem to pass easily from person to person. Dromedary exposure has been shown to be one of the main risk factors for that disease [3,5,6]. The other four coronaviruses that are pathogenic to humans (229E, OC43, NL63, HKU1) are usually associated with mild clinical symptoms [4,7].

According to the International Committee on Taxonomy of Viruses, coronaviruses belong to the subfamily Coronavirinae in the family Coronaviridae [8]. The group of viruses was recognized by a few virologists in 1968 [9]. The viruses were named “coronaviruses” to reflect the characteristic crown-like appearance by which they are identified under an electron microscope (EM) [9]. Coronaviruses are enveloped, positive-sense, single-stranded RNA viruses of pleomorphic shape, measuring between 80 and 160 nm [1,10]. On the basis of serological and genomic evidence, CoVs are categorized into four important genera: *Alphacoronavirus*, *Betacoronavirus*, *Gammacoronavirus*, and *Deltacoronavirus* [1].

Betacoronaviruses have been found mainly in bats [11], camels [12,13], rodents [14] and rabbits [15]. During the past two decades, three known betacoronaviruses have caused epidemics in humans: SARS-CoV, MERS-CoV and SARS-CoV-2 [16]. The SARS-CoV outbreak during 2002–2003 involved 8422 patients and spread to 29 countries globally [17]. A total of 813 fatalities were attributed to that SARS epidemic [1]. Ten years later, MERS-CoV broke out in Saudi Arabia, and later in South Korea, affecting more than 2000 people [18,19].

In December 2019, a few adult patients with pneumonia of unknown cause were admitted to a hospital in Wuhan [20]. In early January 2020, a novel betacoronavirus (SARS-CoV-2, tentatively named 2019-nCoV) was identified from the throat swab sample of a pneumonia patient in Wuhan, followed by many other cases [21,22,23]. The illness caused by SARS-CoV-2 has been officially named Coronavirus Disease 2019 (COVID-19) by the WHO [24,25]. The number of COVID-19 cases has been rising dramatically all over the world, especially in the United States, with the highest rates of death among the elderly and people with comorbidities [26]. The reason for its high infectivity can be found in the unique features of its structural proteins [27] and the capacity of SARS-CoV-2 to be transmitted by asymptomatic people [28,29]. Asymptomatic cases have been mainly reported for infants and children [22,30]. Nonetheless, respiratory droplets are the main route of transmission [7]. Early symptoms in most COVID-19 patients include fever, cough, dyspnea, myalgia, and sore throat [21,31]. A basic reproductive number (R_0_—the expected number of cases directly generated by one case) of SARS-CoV-2 has been determined to range from 2 to 3 [32,33]. However, some studies using mathematical methods estimate the R_0_ of COVID-19 to be as high as 6.49 [34].

This review is focused on the interactions of betacoronaviruses (ß-CoV) with human proteins, with the emphasis on SARS-CoV-2 and its spike (S) structural protein. The importance of mass spectrometry (MS) methods and other instrumental techniques in these studies, which can lead to the results supporting preclinical and clinical studies, has been stressed.

## 2. Differences and Similarities between SARS-CoV and SARS-CoV-2

At present, COVID-19 exhibits less mortality than SARS and MERS; however, it shows much stronger infectivity [35], most likely due to its notable genomic features [27].

In January 2020, Lu et al. revealed 99.98% sequence identity of the viral genomes obtained from patients who were early cases of the outbreak in Wuhan, suggesting a single spill-over event from one source within a very short period [23]. Eight of the nine studied cases had contact with the Huanan seafood market in Wuhan. One patient had never visited the market; however, he had stayed in a hotel near the market before the onset of his disease [23]. 

The newly discovered SARS virus was shown to have a better sequence identity with SARS-CoV than with MERS-CoV [36]. Zhou et al. discovered that SAS-CoV-2 is 96.2% identical at the whole-genome level to a horseshoe bat coronavirus [37]. Additionally, the amino acid sequences of the seven conserved replicase domains in open reading frame (ORF) that were used for coronavirus species classification displayed 94.4% similarity in identity between SARS-CoV-2 and SARS-CoV [37]. This similarity suggests that the two viruses belong to the same species. Consequently, the new coronavirus is thought to be a recombinant virus transmitted from bats to humans by the means of an unknown intermediate host [26]. Some researchers link SARS-CoV-2 to pangolin viruses because of the very high similarity of their receptor-binding domains (RBDs) [38].

The origin of the infection is still under investigation. Two scenarios of the origin of COVID-19 have been proposed: (I) natural selection in viral evolution in an animal host before zoonotic transfer; and (II) natural selection in humans following zoonotic transfer [27]. Some researchers believe that SARS-CoV-2 coronavirus is a recombinant virus between several bat SARS-like betacoronaviruses [39,40].

The RNA genome of coronaviruses is one of the largest among all the RNA viruses. The length of the SARS-CoV genome is 29,727 nucleotides, with 11 open reading frames [41,42]. 

In CoVs, the proteolytic processing of the 5′ two-thirds of the genome results in the production of 16 nonstructural proteins that are involved in viral RNA replication and transcription [43]. Approximately one-third of the betacoronavirus genome encodes four structural proteins: membrane-spanning spike (S), envelope (E) and membrane (M) proteins and a single nucleocapsid (N) protein [10,44] These structural proteins have been shown to play an important role in the pathogenesis and complications of SARS disease [1]. Additionally, the SARS viruses contain a few accessory proteins. Some betacoronaviruses also encode a hemagglutinin esterase (HE) glycoprotein [45]. However, it was shown that SARS-CoV does not encode the HE protein [46]. Interestingly, there is no significant amino acid sequence similarity between the SARS-CoV accessory proteins and any known viral or cellular proteins [47]. Both SARS-CoV and SARS-CoV-2 access their host cells by a process of membrane fusion that is mediated by a homotrimeric “spike” (S) protein [48].

The current knowledge on SARS-CoV-2 is still limited, but every day, new research data become visible. It has been shown that SARS-CoV-2 is distinct from SARS-CoV in the phylogeny of the complete RNA-dependent RNA polymerase (RdRp) gene [23]. 

The most fundamental difference between the SARS-CoV and SARS-CoV-2 viruses lies in their gene sequences. The amino acid sequence of the virus causing COVID-19 differs from other betacoronaviruses. specifically in the regions of the nucleocapsid (N) protein and the S glycoprotein, as well as in the 1ab polyprotein [36]. Among the four structural proteins of SARS coronavirus, the nucleocapsid (N) and the spike (S) have been shown to be the main immunogens [49]. 

Moreover, experiments with UV-inactivated virus revealed that SARS-CoV S protein induces ER stress, which has a significant impact on cell homoeostasis and may contribute to viral pathogenesis [50]. It is worth mentioning that SARS-CoV-2 S glycoprotein shares 97% sequence identity with the spike protein of the bat coronavirus RaTG13 [37,51].

Nucleocapsid protein, with a mass of 50 kDa, is the most abundant macromolecule in coronaviruses and is highly immunogenic [52]. The comparison of the whole amino acid sequence of chosen N proteins revealed 87.86% similarity between SARS-CoV-2 and pangolin CoV, 90% similarity to SARS-CoV, and 99% similarity to bat CoV [52]. The nucleocapsid protein of SARS-CoV has the capacity to neutralize the immune response of the host. If the SARS-CoV-2 N protein does not share that capacity, this can explain the lower mortality rate than that of the 2003/4 SARS epidemic [53]. The spike glycoproteins of SARS coronaviruses are reviewed in more detail below, as well as the current knowledge about their interactions with the human receptor.

Most relevant similarities and differences between SARS-CoV and SARS-CoV-2 have been summarized in Table 1. It should be noted that the estimation of the R_0_ and the mortality rate during the epidemic can be plagued by data uncertainty and variability [54]. 

Very recently, 332 high-confidence SARS-CoV-2 protein–human protein interactions that are associated with multiple biological processes have been identified using affinity-purification mass spectrometry (AP-MS) [70]. Comparison of the interacting proteins between SARS-CoV-2 and ten other pathogens using a hypergeometric test revealed that West Nile virus and *Mycobacterium tuberculosis* had the most similar host-protein interaction partners [70]. The novel SARS virus interactome exposes new aspects of SARS-CoV-2 biology and potential targets for SARS-CoV-2 inhibition.

In another study, MS-based HLA-I (human leukocyte antigens-I) and HLA-II epitope binding prediction tools were utilized to identify SARS-CoV-2 epitopes recognized by helper and cytotoxic T cells (CD4^+^ and CD8^+^, respectively) [71]. Responses to both CD4^+^ and CD8^+^ T cells have been detected in SARS-CoV and in SARS-CoV-2-infected patients [72,73]. Unlike traditional binding assays which rely on chemical synthesis and the a priori knowledge of ligands to be assayed, MS method used by Poran et al. was based on natural peptide-HLA complexes that are subject to the endogenous processing and presentation pathways within the cell [71]. It has been revealed that the relative expression of SARS-CoV-2 proteins in virally infected cells vary significantly; this should be considered in vaccine design to induce cellular immunity [71].

## 3. Spike Glycoproteins

As the S protein is surface-exposed and mediates the host cell penetration by coronaviruses, it is the main focus of vaccine and therapeutic design [48,51]. Understanding the role of the SARS viruses spike glycoprotein and the character of its interaction with host receptor is fundamental to the understanding of viral pathogenesis [74,75]. One of the hypotheses to explain the higher transmission rate of SARS-CoV-2 compared to SARS-CoV is the genetic recombination of the S protein [27,48,76].

**Figure 1:**
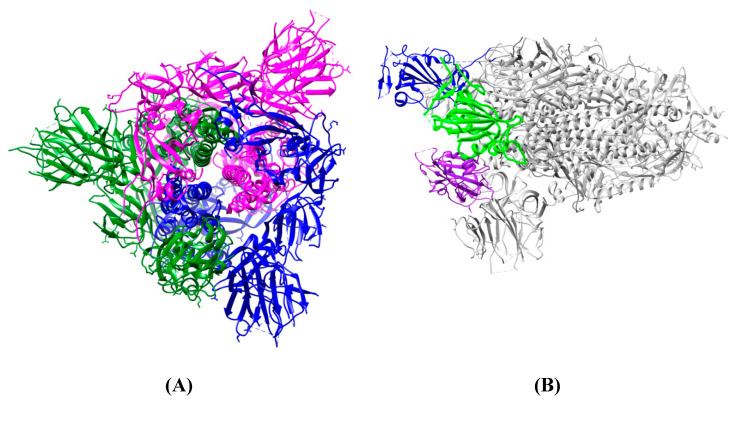

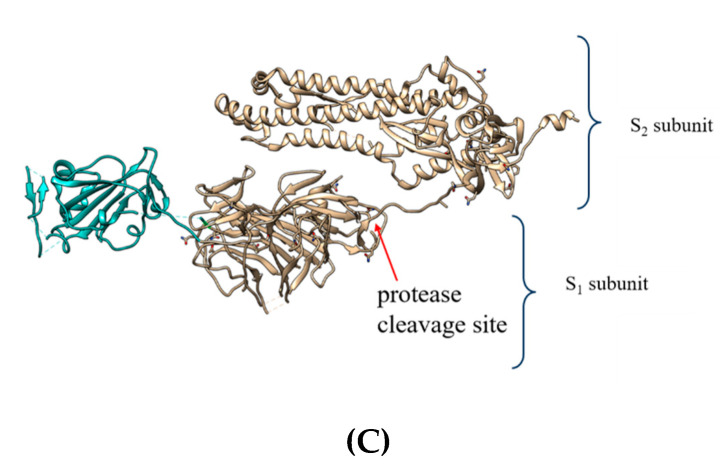
Overall structure of the S protein protomer of SARS-CoV-2. (**A**) Each monomer of the S protein protomer is represented by a different color. (**B**) Receptor-binding sites of the chains A, B, and C of the S protein are highlighted in blue, purple and green color, respectively. Glycan ligands have been omitted for simplicity. (**C**) Monomer of the SARS-CoV2 spike protein during the receptor-accessible conformation. The receptor binding domain (RBD) is shown in cyan. Visualized by USCF Chimera [80]. PDB ID: 6VSB.

The S protein of SARS coronaviruses belongs to class-I viral fusion proteins. It consists of three monomers, each ~1200 amino acid residues long (Figure 1A) [73]. Each monomer of this densely glycosylated spike protein is approximately 180 kDa and contains two subunits, S1 and S2 (Figure 1C), which are responsible for the attachment to the host cell and membrane fusion, respectively [77,78]. S2 subunits of SARS-CoV-2 and SARS-CoV are structurally conserved, whereas the S binding part of SARS-CoV, which is used to recognize its entry receptor, shares only approximately 73–75% overall amino acid sequence identity with SARS-CoV-2 binding domain [79].

Glycosylation is one of the most outstanding post-translational modifications in many viral S or envelop proteins, and some researchers argue that the determination of site-specific glycosylation of virus glycoproteins would enable the development of vaccines that take advantage of glycosylation-dependent mechanisms [81]. Mass spectrometric methods have proved to be very useful for quantifying site-specific glycosylation [81,82]. Indeed, mass spectrometry has arisen as a pivotal method for the characterization of numerous virus surface proteins glycosylation in recent years [83,84].

Although genomic methods are very informative for viral mutation or adaptation through immune selective pressure, they cannot inform on that crucial feature of enveloped viruses—viral spike glycosylation. Exploring spike glycosylation and plasticity with advanced mass spectrometric methods using e.g., recombinant preparations compared to wild type viral proteins can be very helpful for a better understanding of the conformational dynamics that shape receptor or antibody binding [84]. The binding of previous coronavirus S proteins to their respective receptors has been shown by bioinformatics and proteomics approaches to be mediated by its oligomannose N-glycans [82,85]. Very recently, Watanabe et al. revealed, by combined mass spectrometric and cryo-EM analysis, how the N-linked glycans occlude distinct regions across the surface of the SARS-CoV-2 spike protein [86]. To resolve the site-specific glycosylation of the SARS-CoV-2 S protein, three kinds of proteases were used to generate glycopeptides that contain a single N-linked glycan sequon. Liquid chromatography–mass spectrometry (LC-MS) analysis determined the glycan composition for all 22 N-linked glycan sites. It was shown that 8 sites contain substantial populations of oligomannose-type glycans, principally N234 and N709 sites, and the remaining 14 sites are dominated by processed, complex-type glycans [86]. This extensive heterogeneity is similar to this of the S proteins of other coronaviruses such as MERS and HKU1, with the broad distribution of oligomannose-type glycans, without one particular dominant peak, as is the case for some viral glycoproteins [87]. Alteration of glycosites can affect viral infectivity, pathogenesis and host responses, e.g., by sterically masking polypeptide epitopes and modulating S protein-receptor interactions. Distinct epitope features between SARS-RBD and SARS-CoV-2-RBD have been shown by studies using murine polyclonal antibodies [88].

It is worth mentioning that all of the glycan sites are conserved on the S2 subunit between SARS-CoV and SARS-CoV-2, whereas the S1 subunit exhibits glycan site additions and deletions. SARS-CoV-2 maintains a total of 22 N-linked glycan sites in comparison with 23 on SARS, with 18 of these sites being in common [87].

During viral infection, the spike protein is cleaved into these S1 and S2 subunits by nearby host proteases, such as human airway trypsin-like protease (HAT), cathepsins and transmembrane protease serine 2 (TMPRSS2), and releases the signal peptide to promote virus entry into host cells [7,89]. The proteolytic priming event is usually individual; however, SARS coronavirus entry requires another cleavage (on the S2 domain) by the endosomal protease cathepsin [90,91]. That second cleavage activates the protein for the membrane fusion via irreversible conformational changes [92]. 

Recently, it was revealed that SARS-CoV-2 has a furin cleavage site at the boundary between S1 and S2 that has unique sequence among ß-coronaviruses [48]. It had been shown before that the introduction of a furin recognition motif at R667 of SARS-CoV spike glycoprotein allows for efficient cleavage and increased cell-cell fusion activity [93]. The motif of RRAR amino acids in the novel SARS virus, instead of a single arginine, as is present in other similar viruses, allows effective cleavage by furin and other proteases [27,76]. Since furin is highly expressed in lungs, the virus can easily exploit that enzyme to activate its S glycoprotein [76]. Interestingly, Ou et al. discovered that SARS-CoV-2 S protein could trigger syncytia in human receptor cells independently of exogenous protease [77]. The next distinguishing feature of the SARS-CoV-2 spike glycoprotein is the significant variability of its receptor binding domain (RBD). That domain is the most variable part of the coronavirus genome [37,94]. 

**Figure 2:**
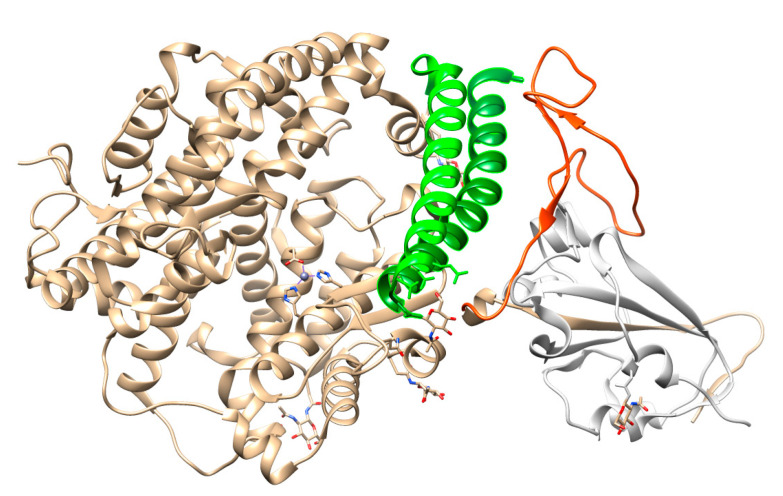
Interactions between SARS-CoV-2 RBD (in orange) and ACE2 (the main interacting α1 helix in dark green, the α2 helix also contributing to the interaction in green), visualized by USCF Chimera. PDB ID: 6M0J.

Different coronaviruses use different domains within the S1 subunit to attach to the appropriate receptor [48]. The RBD of the SARS-CoV-2 spike glycoprotein binds directly to the peptidase domain (PD) of the human cell receptor (Figure 2). These small (~21 kDa each) [51] receptor-binding domains in the whole protomer are depicted in colors in Figure 1B.

Many studies have proved that the human angiotensin-converting enzyme (hACE2) is a functional receptor for SARS-CoV-2, similar to other SARS-related coronaviruses [48,63,95]. Zhou et al. showed that only ACE2, and no other coronavirus receptors, such as aminopeptidase N (APN) and dipeptidyl peptidase 4 (DPP4), are used by SARS-CoV-2 as an entry receptor [37]. However, the primary physiological role of ACE2 is catalyzing the hydrolysis of angiotensin II (a vasoconstrictor peptide) into angiotensin heptapeptide (a vasodilator) [96]. ACE2 is an integral membrane metalloproteinase with the N-terminal extracellular domain containing six canonical sequons for N-linked glycosylation and several potential O-linked sites. The occupancy of N-linked glycans, O-linked glycosylation and the heterogeneity of the O-linked glycans on ACE2 have been recently studied using multiple MS-based approaches, including glycomic and glycoproteomic methods [97]. Glycomic analyses revealed that the majority of ACE2 N-glycans are complex, with limited high-mannose and hybrid glycans. That work with the help of molecular dynamics (MD) simulations revealed crucial roles for glycosylation, not only in sterically masking polypeptide epitopes, but also in directly modulating spike-ACE2 interactions [97].

To engage the ACE2 receptor, the RBD of S1 undergoes hinge-like conformational motions that transiently hide or expose the determinants of receptor binding, as has been shown by Wrapp et al. [51]. These two states are referred to as “up” and “down” conformations, where “up” corresponds to the receptor-accessible state (Figure 1C). Each PD of homodimeric ACE2 protein accommodates one RBD of spike protein, mainly by the arch-shaped α1 helix [63,84]. There is also a limited contribution of the α2 helix, and a loop connecting the ß3 and ß34 strands of ACE2 to that binding (Figure 2).

When the RBD is in the “down” conformation, shielding of receptor binding sites on the SARS-CoV-2 S protein by proximal glycosylation sites (N165, N234, N343) can be observed [86].

Interestingly, two glycans on ACE2 (at N090 and N322) have been predicted by MD to form interactions with the S protein. Each of multiple simulations showed N322 glycan interaction with the S trimer, despite its presence outside of the receptor-binding domain. The arms of the ACE2 glycan at N090 were shown to interact with multiple regions of the S trimer surface over the course of the simulations, exposing the relatively high degree of glycan dynamics [97]. Nevertheless, considerable efforts still need to be overtaken in order to fully understand the role of glycans in SARS-CoV-2 infection and pathogenicity.

In the case of SARS-CoV binding to hACE2, structural work identified 14 positions in RBD that are key for that binding: T^402^, R^426^,Y^436^,Y^440^, Y^442^, L^472^, N^473^, Y^475^, N^479^, Y^484^, T^486^, T^487^, G^488^ and Y^491^ [98]. Analysis of the 144 SARS-CoV-2 genome sequences, done by Walls and co-workers, revealed that 8 out of these 14 positions are strictly conserved, whereas the other 6; R^426^, Y^442^, L^472^, N^479^, Y^484^ and T^487^ (in SARS-CoV) are (semi)conservatively substituted with N^439^, L^455^, F^486^, Q^493^, Q^498^ and N^501^, respectively (in SARS-CoV-2) [48]. 

Yan et al. discovered prominent variations and conformational differences in the interfaces of SARS-CoV and SARS-CoV-2 with the ACE 2 receptor [63]. In that work, the most relevant alteration were shown to be the substitution of Val^404^ in the SARS-CoV-RBD, with Lys^417^ in the RBD of the virus that is responsible for the current pandemic. 

**Figure 3:**
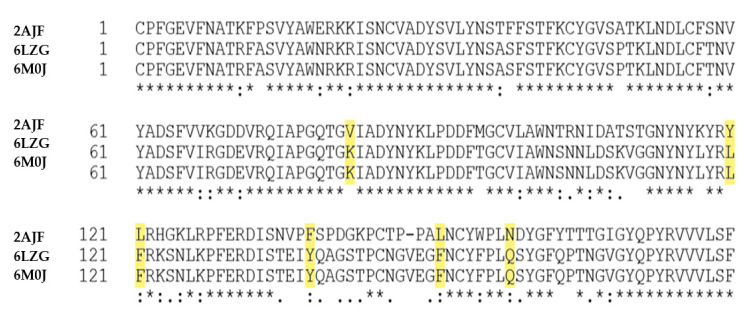
Comparison of the SARS-CoV-RBD/hACE2 and SARS-CoV-2 RBD/hACE2 binding sites. The most prominent substitutions are highlighted in yellow. 2AJF stands for PDB ID of the receptor binding domain of SARS-CoV; below the RBD sequences of the SARS-CoV-2 S1 protein (PDB IDs: 6LZG and 6M0J).

It was suggested that the substitution of other residues (Tyr^442^ to Leu^455^, Leu^443^ to Phe^456^, Phe^460^ to Tyr^473^, and Asn^479^ to Gln^493^) may also influence the affinity for the human cell receptor [63] (highlighted in yellow in Figure 3). Superimposition of the SARS-CoV-2 C-terminal domain (encompassing RBD) structure onto the SARS-RBD structure revealed that the majority of the secondary structure elements are well superimposed in that domain [87]. However, cryo–electron microscopy studies were done in the presence of the neutral amino acid transporter B^0^AT1, which could be the reason for the some divergence of the results compared to the work of Walls et al. [48].

Nonetheless, as shown by Wang et al. among 24 residues in hACE2 that make van der Waals contacts with both RBDs, 15 amino acids exhibit more contacts with the SARS-CoV-2 C terminal domain [87]. Consistently, the SARS-CoV-2 RBD also has more residues than SARS-CoV RBD that directly interact with ACE2, forming vdw contacts and H-bonds. In that work, F486 in SARS-CoV-2, instead of L472 in SARS-CoV, was shown to form strong aromatic-aromatic interactions with Y83 residue of ACE2, and E484 in SARS-CoV-2 instead of P470 in SARS-CoV, formed ionic interactions with receptor’s K31 residue [87].

It has been shown, using biolayer interferometry, that the SARS-CoV-2 S^B^ (binding) domain engages human receptor with comparable affinity to SARS-CoV S^B^ from viral isolates associated with the 2002–2003 epidemic [48]. On the other hand, Wrapp et al. showed that ACE2 binds to the SARS-CoV-2 ectodomain 10- to 20-fold more tightly than to SARS-CoV [51]. Similarly, Wang et al. found that SARS-CoV-2 RBD displays approximately 4-fold stronger affinity towards hACE2 than SARS-RBD. The equilibrium dissociation constant (K_D_) of SARS-CoV-2 RBD binding to ACE2 was calculated to be 133.3 ± 5.6 nM [87]. Surprisingly, computational analyses predicted that the SARS-CoV-2 RBD sequence is not optimal for receptor binding [79].

There are other analytical and biophysical tools which can provide detailed information on the binding affinity of the biomolecules, such as Förster resonance energy transfer (FRET) and biosensors (also FRET-based biosensors) [99,100,101], and isothermal titration calorimetry (ITC) [102,103,104], however, this is not in the scope of this review. The choice of the experimental strategy is usually dictated by the proteins under investigation. Optical biosensors for studies on protein interactions, their advantages and limitations have been thoroughly reviewed by Zhao et al. [105].

The usage of other orthogonal methods supporting MS, X-ray crystallography and cryo-electron microscopy, can give the more detailed insight into the S protein-ACE2 interaction, and find protein targets for the discovery and development of anti-coronavirus therapy [63,99,106].

It is worth mentioning that, five years after the first SARS outbreak, a range of candidate vaccines have been developed. However, as of July 2020, there are no approved vaccines or drugs against any human-infecting CoV infections [48,53,104]. 

Before, despite intensive work on SARS-CoV protease inhibitors, none of the studied compounds have gone through a complete preclinical development program, mainly because of sharp funding cuts in most countries in 2005–2006 [106]. 

There are suggestions that some medicines and vaccines against SARS-CoV could probably have been used to treat infections with the SARS-CoV-2 virus. THis drug repurposing strategy could significantly shorten the time and reduce the cost in comparison to de novo drug discovery and randomized clinical trials [107]. 

However, Wang et al. showed that, despite the structural similarity of RBD of SARS-CoV and SARS-CoV-2, they exhibit different epitope features and differing immunogenicity [86]. On the other hand, Tai and co-workers revealed that SARS-CoV RBD-induced antibodies could cross-react with SARS-CoV-2 RBD and cross-neutralize SARS-CoV-2 pseudovirus infection, suggesting that SARS-CoV RBD-specific antibodies may be used for treatment of SARS-CoV-2 infection [108]. 

More studies are needed to explore the pathogenicity mechanism of SARS-CoV-2, particularly to uncover the mystery of the molecular mechanism of viral entry into host cells and replication. Such studies will provide the basis for future research on developing targeted antiviral drugs and vaccines [109]. 

Since the outbreak at the end of 2019, scientists have been working extensively on therapies and vaccines against the novel coronavirus. Treatments and vaccines not only have to be proven effective against the virus, but must also be safe for people. 

Deeper investigation into the spike protein-human cell receptor/s interactions may provide early scientific guidance for viral prevention and control.

## 4. Conclusions and Future Perspectives

As of 31 August, 25,118,689 cases of COVID-19 have been confirmed in 213 countries and territories around the world. As of that day, approximately 844,000 people have not been able to overcome this disease [110].

The situation with COVID-19 brings the questions: when will it end and what will be next? There is a hypothesis that SARS-CoV-2 will enter the group of the “seasonal infections” in the future [35]. At this moment, despite the many scientific groups working on it, we have only a partial knowledge about this novel virus and its interactions with human cells. Moreover, another adaptive process could result in a virus with even higher infectiousness and transmissibility in humans. 

Furthermore, there are many other species of coronaviruses in animals that can become global health threats. For these reasons, deep studies joining together researchers from the frontiers of biology and chemistry, epidemiologists and doctors are clearly of utmost importance. 

This review provides insights into the COVID-19 current situation, with a special emphasis on the current state of the art in terms of the SARS-CoV-2 S protein and human cell receptor protein interactions. From the lessons learned during the SARS-CoV and SARS-CoV-2 epidemics, we can improve on the handling of global pandemics or epidemics. The clear, reliable and timely dissemination of information is also crucial in dealing with the epidemic. 

Mass spectrometry methods are utilized in studies on virus S protein and receptor protein glycosylation and interaction, revealing targets for neutralizing antibodies elicited through vaccination. Moreover, multi-omic profiling of the host response could be helpful in tracking disease, and prevent future pandemics of similar viruses.

## Figures and Tables

**Table 1:** Comparison of the biological features of SARS-CoV and SARS-CoV-2.

Characteristic	SARS-CoV	SARS-CoV-2	References
Emergence date	November 2002	December 2019	[55,56,57]
Area of emergence	Guangdong, China	Wuhan, China	[55,56,58]
Key hosts	Bat, palm civets, raccoon dogs	Bat, pangolin	[59,60,61]
Entry receptor	ACE2	ACE2	[23,62,63,64]
R_0_	2–5	1.5–6.5	[34]
Transmission	Droplets, contact with infected people	Droplets, contact with infected people, even asymptomatic	[28,29,65]
Mortality rate	9.6%	3–3.6%	[66,67,68]
Symptoms of disease	Fever, headache, cough, dyspnea, shivering, myalgia, malaise and diarrhea	Fever, headache, cough, myalgia, dyspnea, sore throat, chills, and loss of taste or smell, diarrhea	[21,31,57,69]
N protein	IFN-γ inhibitor	Unknown	[35,53]
